# Inverted Pedicled Internal Limiting Membrane Flap Attached to an Optic Disc with Autologous Blood Clot for Large Macular Holes

**DOI:** 10.1155/2023/7640476

**Published:** 2023-07-14

**Authors:** Lishuai Zhang, Huiyu Xi, Jiayu Chen, Aiqin Sheng, Wei Fan, Suyan Li, Haiyang Liu

**Affiliations:** ^1^The Affiliated Xuzhou Municipal Hospital of Xuzhou Medical University, Xuzhou, China; ^2^Department of Ophthalmology, Xuzhou First People's Hospital, Xuzhou, China; ^3^Eye Disease Prevention and Treatment Institute of Xuzhou, Xuzhou, China

## Abstract

**Purpose:**

An inverted ILM flap might be accidentally separated from the retina or sucked away during surgery for large macular holes (MHs). This article is to determine the efficacy of a new inverted pedicled internal limiting membrane (ILM) flap attached to an optic disc with an autologous blood clot (ABC) technique for the treatment of large MHs.

**Methods:**

An inverted pedicled ILM flap connected to the optic disc with ABC was used to treat 12 consecutive patients with significant macular holes (>600 m). The ILM was first peeled off around MH as a semidiameter of about 1.5 diameters of the optic disc. The superior residual ILM was used to produce a pedicled ILM flap that was connected to the optic disc and was later inverted to cover the MH. The macular hole was covered with a repositioned flap larger than 2 MH diameters in an inverted way. ABC was used to fasten the flap, followed by fluid-air exchange with air or C3F8 as tamponade. Spectral domain-optical coherence tomography (SD-OCT) and best-corrected visual acuity (BCVA) were performed at each postoperative follow-up.

**Results:**

The mean aperture and base macular hole diameters were 737.9 ± 109.6 *µ*m (range, 607–982 *µ*m) and 1244.3 ± 227.4 *µ*m (range, 975–1658 *µ*m). All macular holes (100%) were closed after a single surgery without intraoperative or postoperative complications related to the ILM transposition technique. At the last postoperative visit, we found one eye with a U-shaped closure, three eyes with W-shaped closures, and eight eyes with V-shaped closures. No postoperative flap closures were noted in all cases. The preoperative mean BCVA was 1.5 ± 0.3 (range, 1.1–2.0). After a mean follow-up of 5.3 ± 4.8 (range, 3–16) months, the postoperative mean BCVA was 0.8 ± 0.2 (range, 0.6–1.1), and the difference was statistically significant (*p* < 0.05).

**Conclusion:**

This novel technique is safe and suitable for large MHs and can be an alternative option for accidental ILM flap loss during other inverted ILM flap operations.

## 1. Introduction

Pathogenesis and classification of an idiopathic macular hole (IMH) were first expounded by Gass [1], and pars plana vitrectomy (PPV) with gas tamponade was initially introduced to treat MH [2]. Later, vitrectomy combined with internal limiting membrane (ILM) peeling or an ILM flap technique was introduced consecutively to improve the macular hole's anatomical outcomes and visual recovery, especially for those larger MHs [3–5]. As it is known, the inverted ILM flap could provide a scaffold for glial cells and stimulate glial cell proliferation, which would contribute to the hole closure [6–8]. Hence, the inverted ILM flap procedure gradually became the primary surgery to treat MH, significantly for those suffering from macular holes with large diameters (>400 *µ*m), as was suggested by several studies. However, the traditional inverted ILM flap technique has some disadvantages, including the inverted ILM flap being easy to be displaced or sucked away by using a flute needle during the gas-liquid exchange [9–15].

Here, we proposed a new technique that created an inverted pedicled ILM flap attached to the optic disc for covering the MH, and autologous blood clot (ABC) was used to fasten the ILM flap. This new technique may be suitable for large macular holes and also a good choice for accidental ILM flap loss during other inverted ILM flap operations.

## 2. Methods

A total of 12 consecutive patients with large macular holes (>600 *µ*m) were enrolled from April 2019 to November 2021, retrospectively. Recurrent MH was not included. This study was performed according to the Declaration of Helsinki and approved by the Ethics Committee of Xuzhou First People's Hospital (xyy11[2021]-XJSFX-058). Written informed consent was also obtained from all the participants. The associated video (Supplemental Digital Content 1) shows the key steps of the technique procedure.

**Figure 1:**
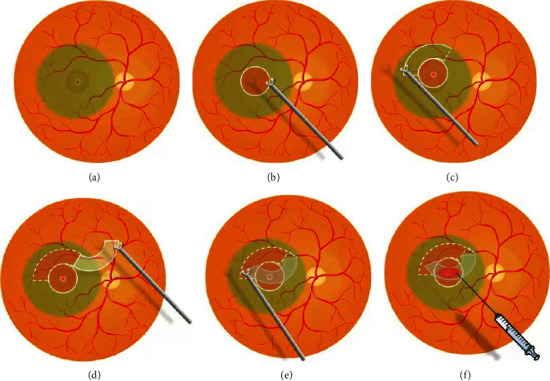
Schematic drawing of the inverted pedicled internal limiting membrane (ILM) flap attached to the optic disc with autologous blood clot (ABC). The illustration of a step-by-step flowchart of the technique. (a) The ILM was stained for 30 seconds with indocyanine green (ICG). (b) ILM around the macular hole (MH) was peeled off. (c, d) A pedicled ILM flap from the superior residual ILM was created with the size larger than a 2 MH area and the root attached to the optic disc. (e) The macular hole was covered with the inverted flap. (f) ABC was used to fasten the flap.

A standard 23-gauge pars plana vitrectomy was performed under retrobulbar anesthesia in all patients by the same surgeon (H.Y.L) using Constellation (Alcon, ForthWorth, TX, USA) under noncontact viewing system Resight 700 (Carl Zeiss Meditec AG, Jena, Germany). In this article, all enrolled patients were combined with a grade II nuclear cataract and received cataract phacoemulsification and IOL implantation. A traditional core vitrectomy with posterior vitreous detachment was performed by triamcinolone-assisted visualization. If present, an epiretinal membrane was peeled after the detached vitreous gel was cleared. The peripheral retina was inspected thoroughly with scleral depression. Laser photocoagulation was applied to these retinal tears or lattices detected during surgery. The ILM was stained with 0.1 ml indocyanine green (ICG, 1.25 mg/ml, Eisai, Inc., Shenyang, China) for 30 seconds. Next, the ILM around the MH was peeled off in an area of 2 to 3 of the size of the optic disc, ensuring the ILM at the edge of the macular hole was removed. An inverted pedicled ILM flap from the superior residual ILM was created with the size larger than a 2 MH area and the root attached to the optic disc. Afterwards, the macular hole was covered with the flap in an inverted way. Finally, ABC was used to fasten the flap, the fluild-air exchange was performed, and C3F8 or air was chosen as tamponade (Figure 1). Patients were instructed to remain in the prone position for about 7–14 days.

Patients were investigated at 14 days, 1 month, and 3 month, postoperatively. At each visit, patients underwent examinations including best-corrected visual acuity or BCVA (logMAR) measurement, slit-lamp examination, fundus examination by using an anterior ophthalmoscope under a slit lamp, and macular imaging with spectral domain-optical coherence tomography (SD-OCT). At each postoperative visit time, the restoration of foveal microstructure, ellipsoid zone (EZ) defects, and external limiting membrane (ELM) defects were estimated by the image of spectral domain-optic coherence tomography.

The restoration of foveal microstructure was described as U-shape, V-shape, W-shape (irregular), flap closure, flat-closure, and flat-open. The first three were considered with satisfactory functional results. Flap closure needed further investigation, which will make improvement to the first three types after several months. The last two types were considered to be associated with poor functional results [16].

## 3. Results

All macular holes (100%) were closed after a single procedure. No intraoperative or postoperative complications related to the ILM transposition technique were noted. In all cases, we succeeded in inverting the flap from the superior retina to cover the MH, with no case spontaneously returning to the original position. No accidental ILM detachment from MH during the fluid-air exchange occurred. The detailed characteristics of patients are summarized in Table 1.

The mean aperture and base macular hole diameters were 737.9 ± 109.6 *µ*m (range, 607–982 *µ*m) and 1244.3 ± 227.4 *µ*m (range, 975–1658 *µ*m). The preoperative mean best-corrected visual acuity was 1.5 ± 0.3 (range, 1.1–2.0). After a mean follow-up of 5.3 ± 4.8 (range, 3–16) months, the postoperative mean best-corrected visual acuity was 0.8 ± 0.2 (range, 0.6–1.1); the difference was statistically significant (*p* < 0.05).

**Figure 2:**
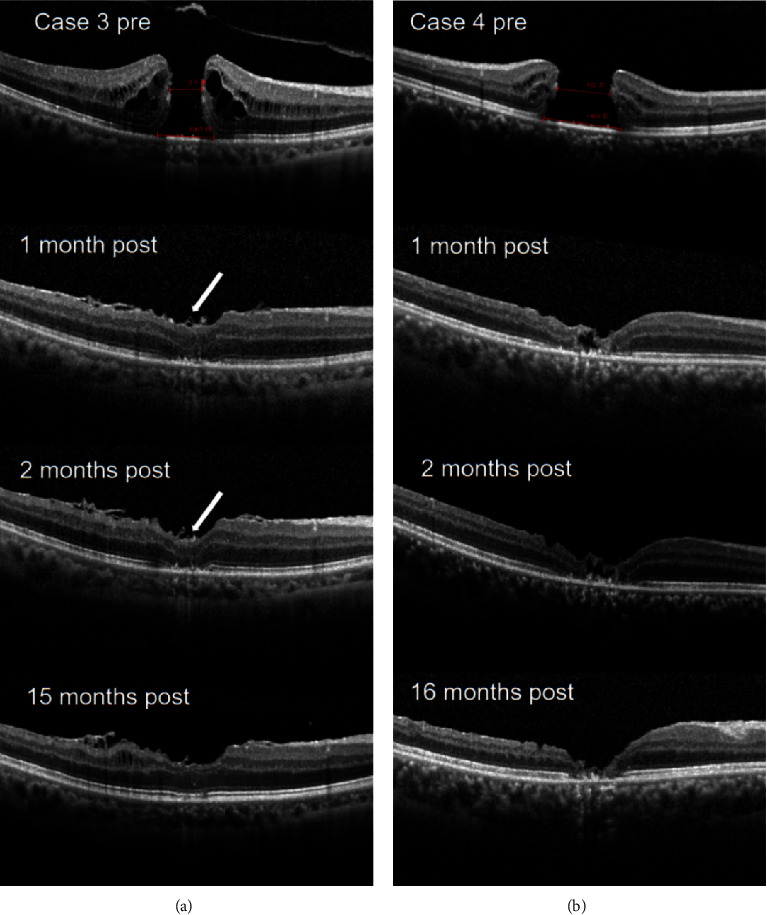
The B-scans of spectral domain-optical coherence tomography (SD-OCT) before and after surgery for case 3 (U-shaped closure) and case 4 (W-shaped closure). (a) The aperture diameter of the macular hole (MH) was 611 *µ*m before surgery. MH closure was obtained, respectively, at the 1st month, 2nd month, and 15th month postoperatively, and the restoration of foveal microstructures was observed. More important is that the intactness of the ellipsoid zone (EZ) and external limiting membrane (ELM) were found at the 15th month postoperatively. The macular hole was covered with a single-layered inverted ILM flap (arrowheads). (b) The aperture diameter of the macular hole (MH) was 982 *µ*m before surgery. MH closure was obtained, respectively, at the 1st month, 2nd month, and 16th month postoperatively, and the incomplete restoration of foveal microstructures was found.

The image of SD-OCT could be clearly obtained 14 days on average postoperatively because of the application of ABC and C3F8 or air. At the last postoperative visit, we found one eye with a U-shaped closure, three eyes with W-shaped closures, and eight eyes with V-shaped closures. No postoperative flap closures were noted in all cases. Representative cases (cases 3 and 4) of the macular hole are presented in Figure 2.

## 4. Discussion

In this new technique, the ILM at the edge of the MH was circumferentially peeled off, and the ILM flap attached to the optic disc lifted from the superior retina was inverted to cover the MH. We considered that this technique is safe and suitable for large MHs and can be an alternative option for accidental ILM flap loss during other inverted ILM flap operations.

**Figure 3:**
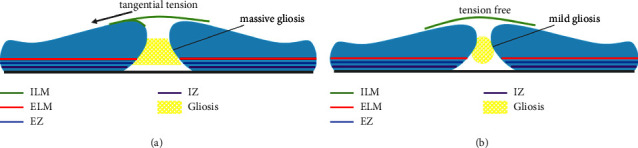
The illustration of the difference in tangential traction between previous techniques and this new technique. (a) When the edge of the macular hole (MH) was selected as the base of the flap, the tangential traction by the ILM could not be released enough, which resulted in massive gliosis. (b) There was no tangential traction by the ILM at the edge of MH of this new technique, which led to mild gliosis. ELM: external limiting membrane, EZ: ellipsoid zone, and IZ: interdigitation zone.

Since Michalewska et al. introduced the inverted internal limiting membrane flap technique in 2010 [3], several modifications have been suggested [3, 9, 17]. All of these were based on the common hypothesis concerning the pathogenesis that the ILM flap provides a scaffold to induce glial cell proliferation and facilitate the MH closure [7, 8]. Photoreceptor cells around MH may move to the fovea on the surface of gliosis to improve visual function [9–15, 17, 18]. However, the classic ILM invert technique selects the edge of the MH as the base of the flap, which might not completely release the tangential traction by the ILM. Thus, a number of large MHs yielded with a flap closure, especially for those with a diameter >600 mm. As reported, the flap closure in the previous studies was found nearly 14%–16% within one month postoperatively, most of which became V-shaped or W-shaped closures after a few months because the macular defects below the inverted ILM flap were filled with gliosis, and a few cases were still flap closures (nearly 3%) after 12 months [6]. Of note, the final BCVA is lower in eyes with an early flap closure than in eyes with initial U-type, V-type, or W-type closures [19, 20]. Theoretically, fewer neuroretinal abnormalities underneath the surgical ILM flap lead to more photoreceptor cells in the fovea, which could yield a better visual outcome [8]. We suspect that the postoperative flap closure could be avoided by releasing the tangential traction of ILM (Figure 3).

A proportion of the surface of ILM around MH contains the residual posterior vitreous cortex to strengthen the tangential traction, so enough release becomes more critical. Concerned about these problems, we modified the technique. In this new technique, the ILM around MH in an area of 2 to 3 of the size of the optic disc as semidiameter is peeled off. The ILM flap attached to the optic disc is lifted from the superior edge of residual ILM. Our new technique combines the benefits of both ILM peeling and ILM flap covering. There was no “flap-closure” case in our report, which is consistent with previous reports, concerning modifying the inverted ILM flap technique along with circumferentially releasing ILM [15, 21]. Moreover, the restoration of foveal microstructures was observed in this study.

Maintaining the stability of the ILM flap was the most challenging part of maneuvering, similar to other ILM flap techniques. In order to improve the retention of the ILM flap-covering MH, key procedures are as follows: First, the ILM flap should be attached to the optic disc, and tight adhesion ensures no free ILM flap. Second, the ILM flap position is transferred from the superior to cover MH, thus avoiding position change because of gravity when the head is upright. Third, ABC was used to fasten the flap. The fresh ABC soon became a clot to cover the macular area after being injected to cover the flap before the fluid-air exchange [22]. Besides, the blood clot is cost-effective, readily available from the patient's antecubital vein, and has extra growth factors to promote MH healing. By applying the abovementioned methods to 12 patients enrolled in this study, our results did not reveal any ILM flap displacement during the fluid-air exchange, proving the effectiveness of the abovementioned methods. In addition, this new technique can be a remedy for accidental ILM flap loss during other inverted ILM flap operations. Sometimes, this is an optional method to treat recurrent MH, with no ILM around MH.

In conclusion, this new surgical technique is safe and effective in treating large macular holes. The advantages of this technique include enough relief of tangential traction around MH and the transposition of the superior pedicled ILM flap to facilitate the MH closure. Long-term follow-up of more patients is needed to confirm the advantage of this technique. Comparable studies are also needed to confirm the superiority of these modifications over the classic inverted ILM flap technique.

## Figures and Tables

**Table 1:** Clinical characteristics of patients who underwent the inverted pedicled ILM flap attached to the optic disc transposition combined with autologous blood clot (ABC).

Case no	Age	Sex	Eye	MH aperture diameter (*µ*m)	MH base diameter (*µ*m)	Highly myopic eye	Intraocular tamponade	Hole closure type	BCVA (logMAR)		Follow-up, months	Complications
									Pre	Post		
1	65	M	Left	607	1626	None	C3F8	W	1.3	0.8	3	None
2	60	F	Right	657	975	None	C3F8	V	1.4	0.7	3	None
3	61	F	Right	611	1063	None	C3F8	U	1.4	0.6	15	None
4	61	F	Right	982	1498	None	C3F8	W	1.1	0.8	16	None
5	65	F	Left	685	1150	None	C3F8	V	1.3	0.7	3	None
6	67	F	Right	779	1195	None	C3F8	V	2	1	5	None
7	57	F	Left	774	1225	None	C3F8	V	1.7	1	3	None
8	65	F	Left	674	1200	None	C3F8	V	1.7	1	3	None
9	69	F	Left	835	1119	None	C3F8	V	1.6	1	3	None
10	63	F	Right	675	1658	None	Air	V	1.1	0.6	3	None
11	70	F	Left	835	1204	None	Air	V	2	1.1	3	None
12	70	F	Left	741	1018	None	C3F8	V	1.4	0.7	4	None

F: female; M: male; MH: macular hole; BCVA: best-corrected visual acuity.

## Data Availability

The datasets used and/or analyzed during the current study are available from the corresponding author on reasonable request.

## Abbreviations

ILM: Internal limiting membrane
IMH: Idiopathic macular hole
SD-OCT: Spectral domain-optical coherence tomography
BCVA: Best-corrected visual acuity
ELM: External limiting membrane
EZ: Ellipsoid zone
ICG: Indocyanine green
logMAR: Logarithm of minimum angle of resolution
ERM: Epiretinal membrane.

## References

[1] Gass J. D. M. (1995). Reappraisal of biomicroscopic classification of stages of development of a macular hole. *American Journal of Ophthalmology*.

[2] Kelly N. E., Wendel R. T. (1991). Vitreous surgery for idiopathic macular holes Results of a pilot study. *Archives of Ophthalmology*.

[3] Michalewska Z., Michalewski J., Adelman R. A., Nawrocki J. (2010). Inverted internal limiting membrane flap technique for large macular holes. *Ophthalmology*.

[4] Park D. W., Sipperley J. O., Sneed S. R., Dugel P. U., Jacobsen J. (1999). Macular hole surgery with internal-limiting membrane peeling and intravitreous air. *Ophthalmology*.

[5] Eckardt C., Eckardt U., Groos S., Luciano L., Reale E. (1997). Entfernung der Membrana limitans interna bei Makulalöchern. *Ophthalmologe*.

[6] Boninska K., Nawrocki J., Michalewska Z. (2018). Mechanism of flap closure after the inverted internal limiting membrane flap technique. *Retina*.

[7] Chou H. D., Liu L., Wang C. T. (2022). Single-layer inverted internal limiting membrane flap versus conventional peel for small or medium-sized full-thickness macular holes. *American Journal of Ophthalmology*.

[8] Shiode Y., Morizane Y., Matoba R. (2017). The role of inverted internal limiting membrane flap in macular hole closure. *Investigative Ophthalmology & Visual Science*.

[9] Andrew N., Chan W. O., Tan M., Ebneter A., Gilhotra J. S. (2016). Modification of the inverted internal limiting membrane flap technique for the treatment of chronic and large macular holes. *Retina*.

[10] Encyclopædia Britannica (2009). *Leaf nosed Bat in Encyclopædia Britannica*.

[11] Michalewska Z., Michalewski J., Dulczewska-Cichecka K., Adelman R. A., Nawrocki J. (2015). temporal inverted internal limiting membrane flap technique versus classic inverted internal limiting membrane flap technique. *Retina*.

[12] Park S. W., Pak K. Y., Park K. H., Kim K. H., Byon I. S., Lee J. E. (2015). Perfluoro-n-octane assisted free internal limiting membrane flap technique for recurrent macular hole. *Retina*.

[13] Hu Z., Ye X., Lv X. (2018). Non-inverted pedicle internal limiting membrane transposition for large macular holes. *Eye*.

[14] Tian T., Chen C., Peng J., Jin H., Zhang L., Zhao P. (2019). Novel surgical technique of peeled internal limiting membrane reposition for idiopathic macular holes. *Retina*.

[15] Hu Z., Qian H., Fransisca S. (2020). Minimal internal limiting membrane peeling with ILM flap technique for idiopathic macular holes: a preliminary study. *BMC Ophthalmology*.

[16] Kang S. W., Ahn K., Ham D. I. (2003). Types of macular hole closure and their clinical implications. *British Journal of Ophthalmology*.

[17] Shin M. K., Park K. H., Park S. W., Byon I. S., Lee J. E. (2014). Perfluoro-n-octane-assistedsingle-layered inverted internal limiting membrane flap technique for macular hole surgery. *Retina*.

[18] Fung N. S. K., Mak A. K. H., Yiu R., Wong I. Y. H., Lam W. C. (2020). Treatment of large, chronic and persistent macular hole with internal limiting membrane transposition and tuck technique. *International Journal of Retina and Vitreous*.

[19] Jen P. H. S., Wu C. H. (2008). Echo duration selectivity of the bat varies with pulse-echo amplitude difference. *NeuroReport*.

[20] Tsui M. C., Yang C. M. (2021). Early and late macular changes after the inverted internal limiting membrane flap technique for a full-thickness macular hole. *Retina*.

[21] Greenhall A. M. (1982). *House Bat Management in, Northern Prairie Wildlife Research Center Online*.

[22] Wu A. L., Chuang L. H., Wang N. K. (2018). Refractory macular hole repaired by autologous retinal graft and blood clot. *BMC Ophthalmology*.

